# New-York Hotels

**Published:** 1860-01

**Authors:** 


### NEW-YORK HOTELS.

For the convenience of our readers, who are scattered all over every where, and who may chance to come to the metropolis of the nation, for purposes of business, health, or pleasure, the notice below is given from the *Home Journal*, which for elegance in manner and matter may be considered the first and best weekly publication of its kind, not only in the Union, but in the world. In transferring the article to the pages of the Journal of Health, it is premised that our office and residence are within three blocks of Union Place Hotel, the Everett House, and the Clarendon. Consequently we are very near royalty, for we have long observed that titled persons from abroad oftener "put up" at one of the three houses named than elsewhere, for true nobility always seeks retiracy, quiet, and comfort. We are not personally acquainted with either of the "hosts," and make this mention less for them than for our readers.

"Hotel life is so rapidly superseding house-keeping in this metropolis, that the private residence promises soon to become a rarity; and indeed this is not strange when we consider the exorbitant rents demanded for decent tenements, and contrast the comforts of our first-class hotels and their well-drilled attendants, with our ten-pin-alley houses all up-stairs, and the stupid servants that so try the patience of housekeepers. The hotels of this city have attained such size and magnificence, as to be among the curiosities for country visitors to see. Foreigners are especially surprised at their luxury and elegance, and have taken many valuable hints from their management and arrangements. The old Astor still continues to be a favorite among the merchants whose duties require proximity to their counting-houses, and maintains its reputation for prompt attendance and bountiful larder. The St. Nicholas is patronized principally by transient visitors, and persons fond of the excitement inseparable from large crowds. The Metropolitan is popular on account of its central locality, being near the shops of Broadway, and most of the places of public amusement. The New-York Hotel is remarkable for the artistic marvels of the *entres;* and the Brevoort, for the privacy of its suites of rooms and the excellent family restaurant. The Fifth Avenue Hotel has been filled since the first day of its opening, and seems to give satisfaction. A new house always has a rush, and its popularity afterwards is dependent upon its management. The Everett is generally preferred by those who remain for any length of time in town, on account of its beautiful locality—being opposite Union Square, on the corner of Seventeenth street and Fourth Avenue—and the admirable management of all the interior arrangements, in order to afford every attainable comfort and enjoyment for its inmates. Its recent proprietor, Mr. Clapp, has retired from business, and is succeeded by Mr. L. L. Britton, who has had much experience in keeping hotels, having been for twelve years the proprietor of the best house in Albany. That the excellence of the Everett will be sustained under the new government, there is every reason to believe. Mr. Britton is making a few changes that will be very acceptable and attractive. The admirable Union Place Hotel, the St. Denis, the La Farge, the Clarendon, the Prescott, and other well-known establishments, are, like those already mentioned, generally well filled with visitors. This is also the case with the hundreds of hotels "*on the European plan*," and the thousands of boarding-houses *on no plan at all*, which are scattered every where throughout the city. A monster hotel on Fifth Avenue, opposite the Central Park, to occupy an entire square, is in contemplation. It will, of course, make a sensation—when finished."

